# Donor-Matched Comparison of Chondrogenic Potential of Equine Bone Marrow- and Synovial Fluid-Derived Mesenchymal Stem Cells: Implications for Cartilage Tissue Regeneration

**DOI:** 10.3389/fvets.2016.00121

**Published:** 2017-01-18

**Authors:** Mohammed Zayed, Christopher Caniglia, Nabil Misk, Madhu S. Dhar

**Affiliations:** ^1^Department of Large Animal Clinical Sciences, College of Veterinary Medicine, University of Tennessee, Knoxville, TN, USA; ^2^Department of Animal Surgery, College of Veterinary Medicine, South Valley University, Qena, Egypt; ^3^Manor Equine Hospital, Monkton, MD, USA; ^4^Department of Animal Surgery, College of Veterinary Medicine, Assiut University, Asyut, Egypt

**Keywords:** chondrogenesis, mesenchymal stem cells, bone marrow, synovial fluid, cartilage regeneration, horse

## Abstract

Mesenchymal stem cells (MSCs) have been demonstrated to be useful for cartilage tissue regeneration. Bone marrow (BM) and synovial fluid (SF) are promising sources for MSCs to be used in cartilage regeneration. In order to improve the clinical outcomes, it is recommended that prior to clinical use, the cellular properties and, specifically, their chondrogenic potential must be investigated. The purpose of this study is to compare and better understand the *in vitro* chondrogenic potential of equine bone marrow-derived mesenchymal stem cells (BMMSCs) and synovial fluid-derived mesenchymal stem cells (SFMSCs) populated from the same equine donor. BM- and SF-derived MSCs cultures were generated from five equine donors, and the MSCs were evaluated *in vitro* for their morphology, proliferation, trilineage differentiation, and immunophenotyping. Differences in their chondrogenic potentials were further evaluated quantitatively using glycosaminoglycan (GAG) content and *via* immunofluorescence of chondrogenic differentiation protein markers, SRY-type HMG box9, Aggrecan, and collagen II. The BMMSCs and SFMSCs were similar in cellular morphology, viability, and immunophenotype, but, varied in their chondrogenic potential, and expression of the key chondrogenic proteins. The SFMSCs exhibited a significant increase in GAG content compared to the BMMSCs (*P* < 0.0001) in three donors, suggesting increased levels of chondrogenesis. The expression of the key chondrogenic proteins correlated positively with the GAG content, suggesting that the differentiation process is dependent on the expression of the target proteins in these three donors. Our findings suggest that even though SFMSCs were hypothesized to be more chondrogenic relative to BMMSCs, there was considerable donor-to-donor variation in the primary cultures of MSCs which can significantly affect their downstream application.

## Introduction

Osteoarthritis (OA) is a chronic disease pertaining to progressive deterioration of the articular cartilage and subchondral bone (1). OA is a major cause of reduced performance and loss in human and equine population (2–4). Attempts have been made to treat OA and prevent joint degeneration, which primarily includes injection of pain relief medications to reduce inflammation and treat pain (5). Since, cartilage degeneration is a sequel to OA, aggressive surgical approaches such as chondrocyte implantation have also been used in the treatment of massive chondral injuries in human and equine patients (6, 7). To date, no therapies are available to effectively regenerate the affected tissue, posing OA as a major health concern. In recent years, mesenchymal stem cells (MSCs) have shown promise in human and equine regenerative medicine and have been used in tissue regeneration and treatment of many diseases including those affecting the joint (8–12).

Equine adult MSCs are multipotent progenitor cells that can be isolated from various adult tissues, including bone marrow (BM), adipose tissue (AD), and peripheral blood (13–16). Recently, MSCs generated from equine synovial fluid (SF) have been shown to be multipotent *in vitro* (17, 18). Even though MSCs can be easily isolated from various sources, researchers have reported differences regarding their basic biological properties, *in vitro*. These differences include proliferation, differentiation, and immunomodulatory effect, which can adversely impact their function and hence, the *in vivo* application (19–21). Furthermore, variations or differences can occur in the properties of MSCs generated not only from different sources but also from the same source in different donors. For instance, we reported a study where we observed differences within bone marrow-derived mesenchymal stem cells (BMMSCs) in a group of age- and gender-matched horses (22).

Bone marrow is an acceptable adult stem cell source used in cartilage tissue engineering (23, 24). However, possible contamination during BM aspiration, decrease in proliferation and chondrogenic potential with age, hypertrophy, and mineralization when implanted *in vivo* hinder its clinical application (25). Hence, BMMSCs may not be the best source for cartilage repair. Recently, it has been recognized in both human and equine medicine that SF collection is relatively easy, less invasive, and a rich source of MSCs. In addition, the synovial MSCs have greater proliferative and chondrogenic abilities than other MSCs, making SF or the synovium cells as an alternative, accessible source of MSCs and a suitable candidate for cartilage repair (17, 26, 27).

Although BM and SF represent attractive tissue sources for MSCs, there has been no comparison of the chondrogenic potential of these cells from the same donor. Based on published papers and the results from our laboratory, we hypothesize that equine synovial fluid-derived mesenchymal stem cells (SFMSCs) can be isolated and characterized *in vitro* and their chondrocyte differentiation would be superior to BMMSCs, but there may be donor-to-donor variations when donor-matched comparison is done. In order to prove our hypothesis, we performed a donor-matched comparison of the biological properties of equine MSCs isolated from BM and SF. Differences, if any, in their chondrogenic potentials were evaluated using glycosaminoglycan (GAG) assay and by assessing changes in the key proteins of chondrogenic differentiation using immunofluorescence (IF).

## Materials and Methods

### Tissue Harvest and Isolation of MSCs

All the experiments were conducted in accordance with the protocols approved by University of Tennessee Institutional Animal Care and Use Committee. Five healthy, mixed breed horses, aged 8–13 years were used for BM and SF collections.

#### Bone Marrow

All procedures were performed as described previously (22, 28). Mononuclear fraction of cells from BM (*n* = 5) collected in the presence of 100 IU/ml heparin (Celsus, Cincinnati, OH, USA) were seeded in the basal medium consisting of Dulbecco’s Modified Eagle Medium/Ham’s F-12 (Fisher Scientific, Pittsburgh, PA, USA), 10% fetal bovine serum (Merck Animal Health, Summit, NJ, USA), and 1% penicillin/streptomycin solution (Fisher Scientific, Pittsburgh, PA, USA) and incubated at 37°C, with 5% CO_2_ (Fisher Scientific, Pittsburgh, PA, USA). Adherent cells were allowed to reach 70–80% confluency before being harvested with 0.25% trypsin EDTA (Fisher Scientific, Pittsburgh, PA, USA) for 2 min at 37°C.

#### Synovial Fluid

Synovial fluid (*n* = 5) was obtained using methods described previously (17, 18) with slight modifications. Horses were sedated with detomidine HCl (Pfizer Animal Health, NY, USA) (0.015–0.26 mg/kg bwt i.v.), 2–5 ml of SF was collected aseptically from each of the radiocarpal, intercarpal, and tarsocrural joints. Approximately 0.5 ml of each sample was collected in microtainer tubes containing EDTA (Fisher Scientific, Pittsburgh, PA, USA) and was analyzed for cytology. For proliferation and expansion of SFMSCs from confirmed clinically normal SF, 1 ml of SF was diluted (1:10) with DMEM F-12 (Fisher Scientific, Pittsburgh, PA, USA) basal medium and seeded onto 75 cm^2^ vented tissue culture flasks (Fisher Scientific, Pittsburgh, PA, USA) and incubated at 37°C, with 5% CO_2_ (Fisher Scientific, Pittsburgh, PA, USA) for 4 days before the first medium change. Thereafter, medium was changed every 2–3 days until the cells reached 70–80% confluency. Adherent cells were harvested with 0.25% trypsin EDTA (Fisher Scientific, Pittsburgh, PA, USA) for 1 min at 37°C.

Each horse was sampled twice between 3 and 6 months. All the cell culture experiments were carried out in triplicate, and each assay was repeated with the equine MSCs from two independent BM and SF harvests.

### Nuclear/Cytoplasmic Staining

Nuclear/cytoplasmic fluorescent staining was used to demonstrate the morphology of MSCs for cytoplasmic staining; MSCs were stained with 5 µg of WGA (Life Technologies, Grand Island, NY, USA) (wheat germ agglutinin, Alexa Fluor 488 conjugate) for 10 min. To stain the nucleus, cells were further washed and stained with 5 µg of TO-PRO-3 iodide stain (Life Technologies, Grand Island, NY, USA) for 10 min. After washing, the cells were mounted with Slow Fade Gold Antifade Reagent (Life Technologies, Grand Island, NY, USA), and images were obtained using a laser scanning spectral confocal microscope (Leica Microsystems©, Wetzlar, Germany).

### Cell Viability and Proliferation

The cellular viability and proliferation of 2.0 × 10^4^ cells of P2–P3 of expanded equine BMMSCs and SFMSCs from each donor (*n* = 5) was assessed at 2, 4, and 8 days using the CellTiter 96 Aqueous Non-Radioactive (MTS) assay (Promega, Madison, WI, USA), as described previously (20, 22). The optical density of the cell and the MTS reagent complex was measured on a microplate fluorescence reader (BioTek, Winooski, VT, USA) at 490 nm. Medium without cells was used as a blank. Graph of absorbance at 490 nm vs. days of proliferation was generated.

### Immunophenotyping

Flow cytometric analysis was performed in P2–P3 expanded BMMSCs and SFMSCs from each donor (*n* = 5) using a panel of cluster-of-differentiation (CD) antigen antibodies. Briefly, for each staining, 1 × 10^6^ cells were harvested and collected in 1× florescence-activated cell sorting (FACS) buffer (phosphate-buffered saline with 0.1% bovine serum albumin) (Fisher Scientific, Pittsburgh, PA, USA). The Fc receptors were blocked by preincubating cells with 0.5 mg/ml mouse (BD Bioscience, Grand Island, NY, USA) Fc block for 20 min on ice. After repeated washing, cells were incubated with fluorescein isothiocyanate (FITC) preconjugated CD90 (BD Bioscience, Grand Island, NY, USA), CD29 (Beckman Coulter, Brea, CA, USA), CD34 (Millipore, Billerica, MA, USA), major histocompatibility complex II (MHC-II) (AbD Serotec, Raleigh, NC, USA), and non-conjugated CD44 (Abcam, Cambridge, MI, USA) primary antibodies for 60 min in the dark on ice. To detect CD44, cells were stained with the secondary antibody for 30 min on ice (Table 1). Negative control staining was performed using a FITC conjugated mouse IgG1 (BD Bioscience, Grand Island, NY, USA) isotype. The raw data of fluorescence were measured using the BD FACS Diva software (BD Bioscience, Grand Island, NY, USA). The percentages of cells positive for a given protein were recorded.

**Table 1:** **Antibodies used for analyzing the specific molecular markers on the cell surface**.

Protein marker	Clone	Dilution	Form
CD90	OX-7	1:10	Fluorescein isothiocyanate (FITC) conjugate
CD34	581	1:5	FITC conjugate
CD29	4B4LDC9LDH8	1:5	FITC conjugate
CD44	IM7	1:100	Non-conjugate
Major histocompatibility complex II monomorphic	CVS20	1:10	FITC conjugate
Secondary (FITC)	–	1:100	Alexa Fluor 488 anti-rat
Isotype control (IgG1)	A85-1	1:10	FITC conjugate

### Adipogenesis and Osteogenesis

Roughly, 2.0 × 10^5^ BMMSCs and SFMSCs from each donor (*n* = 5) were induced to adipogenesis and osteogenesis. Cells were seeded in tissue culture dishes in the basal medium, with the medium change every 2–3 days. At 70–80% confluency, cells were induced to differentiate into each of the two lineages using lineage-specific differentiation media. Adipogenic differentiation was induced in complete growth medium supplemented with 1 µmol/l dexamethasone (BioTek, Winooski, VT, USA), 10 µg/ml recombinant human insulin (BioTek, Winooski, VT, USA), 0.5 mmol/l 3-isobutyl-1-methylxanthine (BioTek, Winooski, VT, USA), 15% rabbit serum (BioTek, Winooski, VT, USA), and 20 µmol/l indomethacin (BioTek, Winooski, VT, USA). Osteogenic differentiation was induced in complete growth medium supplemented with 100 nmol/l dexamethasone, 10 mmol/l β-glycerophosphate (BioTek, Winooski, VT, USA), and 0.25 mmol/l ascorbic acid (BioTek, Winooski, VT, USA). Adipogenic and osteogenic differentiation was confirmed at day 7 and 14 using 0.5% Oil Red O staining and 2% Alizarin Red S staining (Fisher Scientific, Pittsburgh, PA, USA), respectively. Stained cells were visualized and photographed using Nikon DS-Fi2 connected to a Zeiss microscope and evaluated using the NIS Elements imaging software (BioTek, Winooski, VT, USA).

### Chondrogenesis and GAG Content

A total of 70–80% confluent 2.0 × 10^5^ BMMSCs and SFMSCs from each donor (*n* = 5) were induced to chondrogenic differentiation. Differentiation was induced using complete growth medium supplemented with 100 nmol/l dexamethasone, 0.25 mmol/l ascorbic acid (Sigma-Aldrich, Saint Louis, MI, USA), and 5 ng/ml transforming growth factor beta (R&D Systems, Minneapolis, MI, USA) for 14 days. To confirm chondrogenic differentiation, cells were stained with Alcian Blue (pH = 2.5) (Fisher Scientific, Pittsburgh, PA, USA). To measure the GAG content, subsequent to staining, Alcian Blue dye was extracted by exposing the cells to 500 µl of 6M guanidine/HCl (Fisher Scientific, Pittsburgh, PA, USA) overnight with shaking at room temperature. The optical density of the extracted Alcian Blue was measured on a microplate fluorescence reader (BioTek, Winooski, VT, USA) at 620 nm.

### IF Staining

Bone marrow-derived mesenchymal stem cells and SFMSCs from each donor (*n* = 5) were cultured as described above until 70–80% confluent and were subsequently induced to differentiate into chondrocytes using the cocktail described above for 14 days. Differentiated cells were fixed with 4% paraformaldehyde for 10 min, permeabilized with 0.1% Triton X-100 (Sigma-Aldrich, Saint Louis, MI, USA) for 10 min, and finally blocked with 1% Power Block (BioGenex, San Ramon, CA, USA) for 30 min. For each analysis, SRY-type HMG box9 (SOX9) (Santa Cruz Biotechnology, Dallas, TX, USA), Aggrecan (Fisher Scientific, Pittsburgh, PA, USA), and collagen type II (Col II) (Abcam, Cambridge, MI, USA) were used to detect the target proteins (Table 2). Antigen detection was performed using specific secondary antibodies. Samples were mounted in Prolong Gold antifade reagent with DAPI (Life Technologies, Grand Island, NY, USA), and images were obtained with a laser scanning spectral confocal microscope (Leica Microsystems©, Wetzlar, Germany). Fluorescent-labeled secondary antibodies alone and FITC-labeled anti-mouse immunoglobulin G1 were used as negative controls. Mean intensity fluorescence was quantified using the Leica TCS SP2 software by selecting at least four representative areas of the same settings.

**Table 2:** **Differentiation time, dilution, and secondary antibodies for chondrogenic protein markers using immunofluorescence analysis**.

Protein marker	Differentiation day	Dilution	Concentration (µg/ml)	Secondary antibody
SRY-type HMG box9	3	1:100	2	Alexa fluor 647 anti-rabbit
Aggrecan	14	1:200	4	Alexa Fluor 488 anti-mouse
Collagen type II	14	1:200	4	Alexa Fluor 488 anti-rabbit

### Statistical Analysis

For the statistical analysis, all quantitative group data are shown as the mean ± SD. Data for cell proliferation, GAG content, immunophenotyping, and IF from the two cell sources were analyzed and compared using Student’s *t*-test (SPSS version 23, SPSS, Chicago, IL, USA). Differences of *P* < 0.05 were considered to be statistically significant.

## Results

### Isolation and *In Vitro* Expansion of MSCs

**Figure 1:**
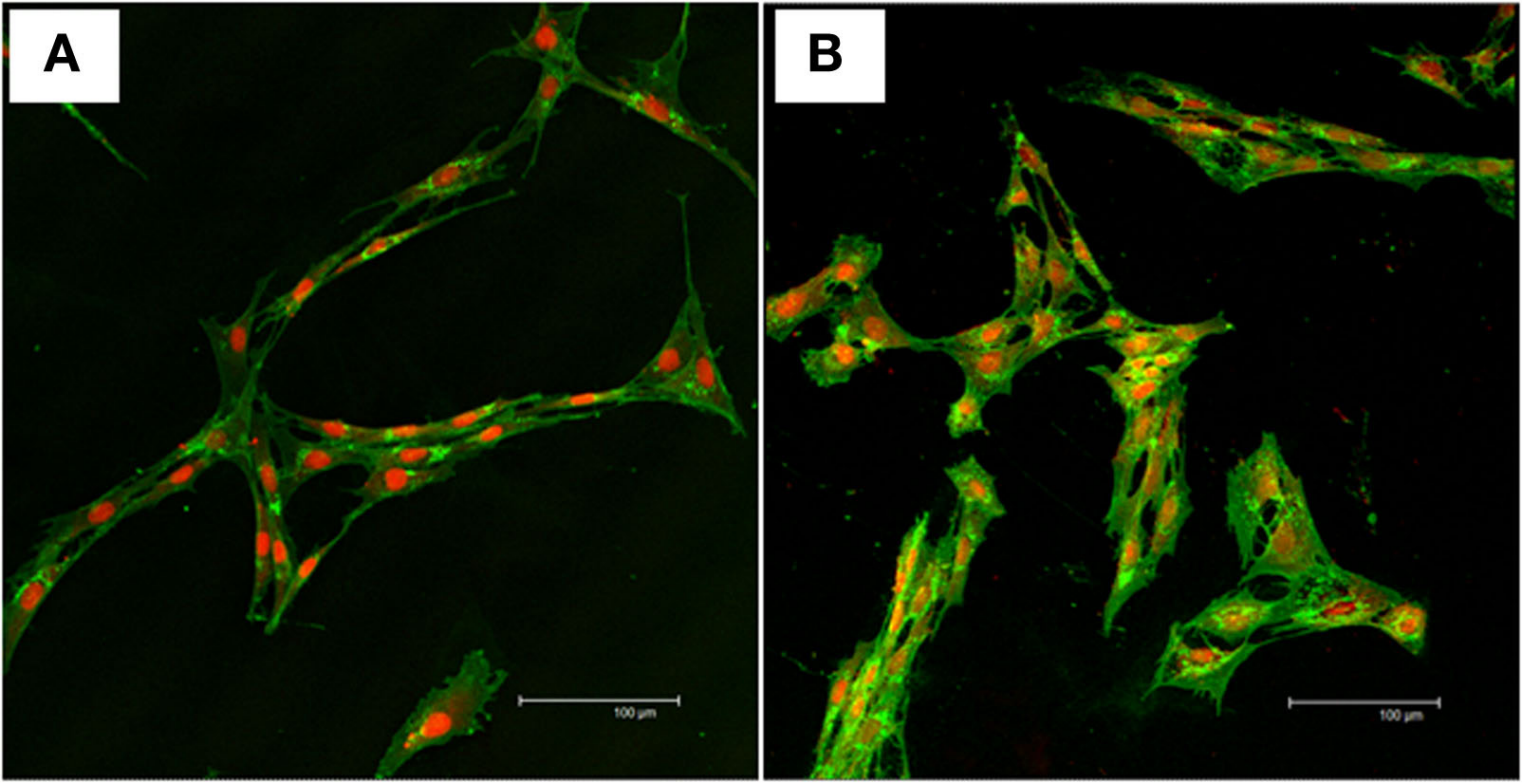
**Nuclear/cytoplasmic staining of mesenchymal stem cells**. Representative confocal images of WGA (green) and TOPRO-3-iodide (red) fluorescent stains showing the viability and morphology of the cells. **(A)** Typical fibroblast-like morphology of undifferentiated bone marrow-derived mesenchymal stem cells. **(B)** Typical fibroblast-like morphology of undifferentiated synovial fluid-derived mesenchymal stem cells. Scale bar = 100 µm.

Bone marrow-derived mesenchymal stem cells adhered to the tissue culture polystyrene-treated plastic within 48 h from seeding, proliferated, and maintained spindle-like morphology throughout the expansion process. The SFMSCs were rounded when seeded in culture, acquired spindle-like morphology within 4 days after seeding, proliferated, and maintained spindle-like morphology throughout the expansion process. The cellular morphology was monitored and observed up to passage 6 for both cell types. The morphology was confirmed using a standard nuclear/cytoplasmic fluorescent staining (Figures 1A,B).

### Cell Proliferation and Viability

**Figure 2:**
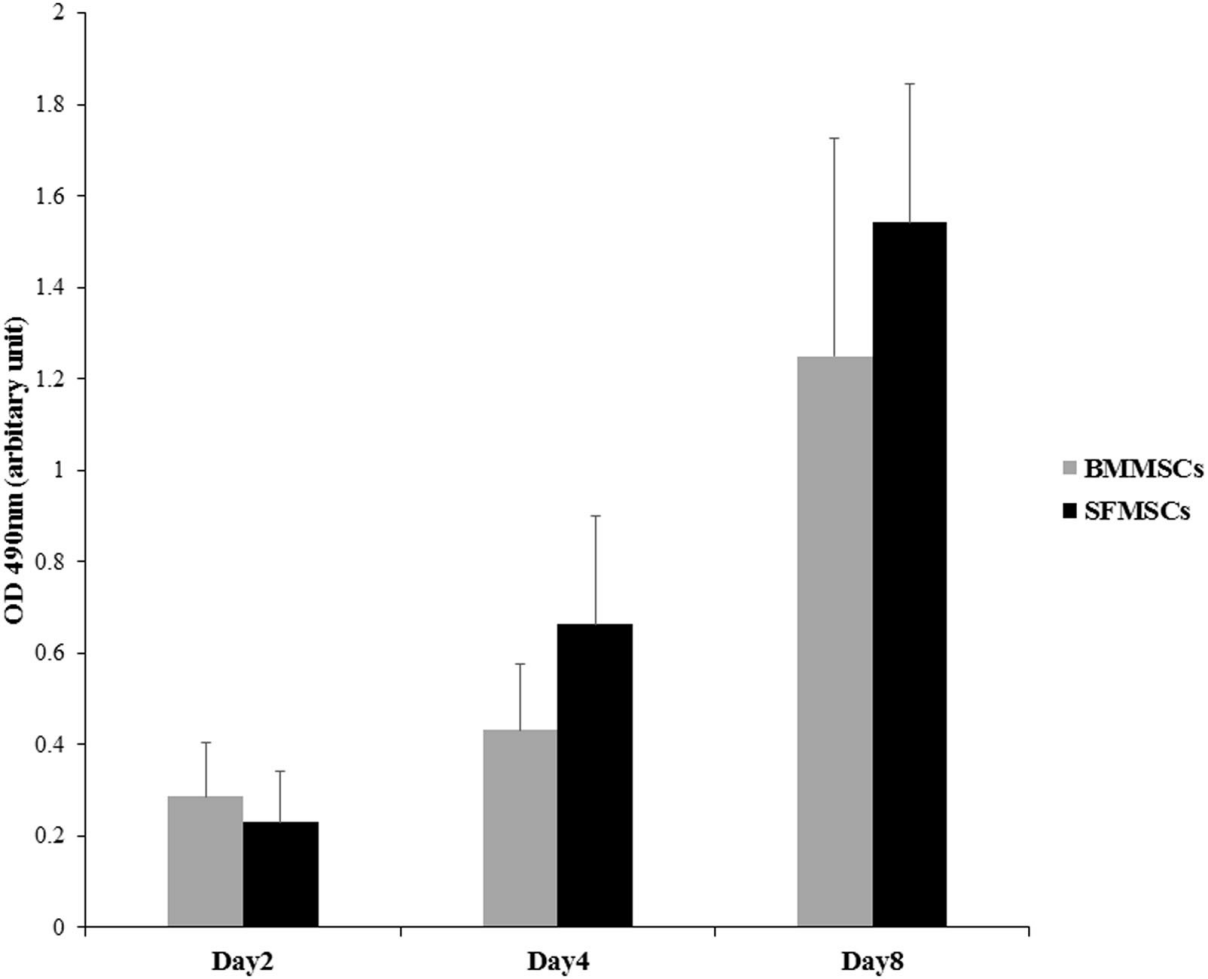
**Measurement of cell proliferation of bone marrow-derived mesenchymal stem cells (BMMSCs) and synovial fluid-derived mesenchymal stem cells (SFMSCs)**. MTS assay was used to evaluate the rate of proliferation over a period of 8 days. Absorbance, optical density at 490 nm is linearly related to the cell numbers and hence represents the proliferation at a given time point. Increase in the optical density between two time points can be used as an index to measure the rate of proliferation. Data are presented as means ± SD. Error bars represent the SD.

Cellular proliferation and viability of BMMSCs and SFMSCs were assessed through a period of 8 days using MTS assay (Figure 2). In presence of the growth medium provided, both cell types were viable and the cell numbers increased linearly over time, suggesting that both types of MSCs can be expanded *in vitro* to generate sufficient numbers for implantation.

### Immunophenotyping

Flow cytometric analyses of BMMSCs and SFMSCs from all donors, revealed >70% expression of MSCs markers including CD29, CD44, and CD90. The BMMSCs were negative for CD34, a hematopoietic marker, and MHC-II. Comparatively, the SFMSCs exhibited low levels of CD34 and significantly high expression of MHC-II. The average percentage of each marker with SD is illustrated in Table 3.

**Table 3:** **Percentage means of mesenchymal stem cells (MSCs) from bone marrow (BM) and synovial fluid (SF) of the same donor for the CD90, CD29, CD44, CD34, and major histocompatibility complex II (MHC-II) markers by flow cytometry**.

Donors	CD90	CD29	CD44	CD34	MHC-II
Bone marrow-derived mesenchymal stem cells (BMMSCs) donor 1	95.55 ± 0.55	75.6 ± 3	96.6 ± 0	1.15 ± 0.85	0.28 ± 0
Synovial fluid-derived mesenchymal stem cells (SFMSCs) donor 1	83.35 ± 2.35	90.55 ± 1.65	91.5 ± 4.5	0.95 ± 0.75	3.245 ± 2.65
BMMSCs donor 2	90.95 ± 4.35	65.95 ± 7.3	99.1 ± 0	1.05 ± 0.05	1.36 ± 0.36
SFMSCs donor 2	76.3 ± 6	71.55 ± 6.75	94.25 ± 1.25	1.45 ± 0.35	7.805 ± 5.1
BMMSCs donor 3	97.25 ± 2.55	86.35 ± 12.15	92.65 ± 7.3	0.635 ± 0.36	1.85 ± 0.45
SFMSCs donor 3	98.9 ± 0.5	99.75 ± 0.25	99.9 ± 0	10.25 ± 0.25	10.2 ± 0.8
BMMSCs donor 4	86.15 ± 0.65	98.8 ± 0.85	99.9 ± 0.1	0.15 ± 0.05	1.75 ± 0.65
SFMSCs donor 4	95.3 ± 0.1	98.8 ± 0.8	99.4 ± 0.4	1.72 ± 0.02	6.15 ± 1.4
BMMSCs donor 5	99.9 ± 0.05	99.6 ± 0.1	99.8 ± 0	3.5 ± 0.3	4.1 ± 0.9
SFMSCs donor 5	96.4 ± 2.8	99.4 ± 0.1	99.7 ± 0.05	0.99 ± 0.5	21 ± 6

### Adipogenesis and Osteogenesis

**Figure 3:**
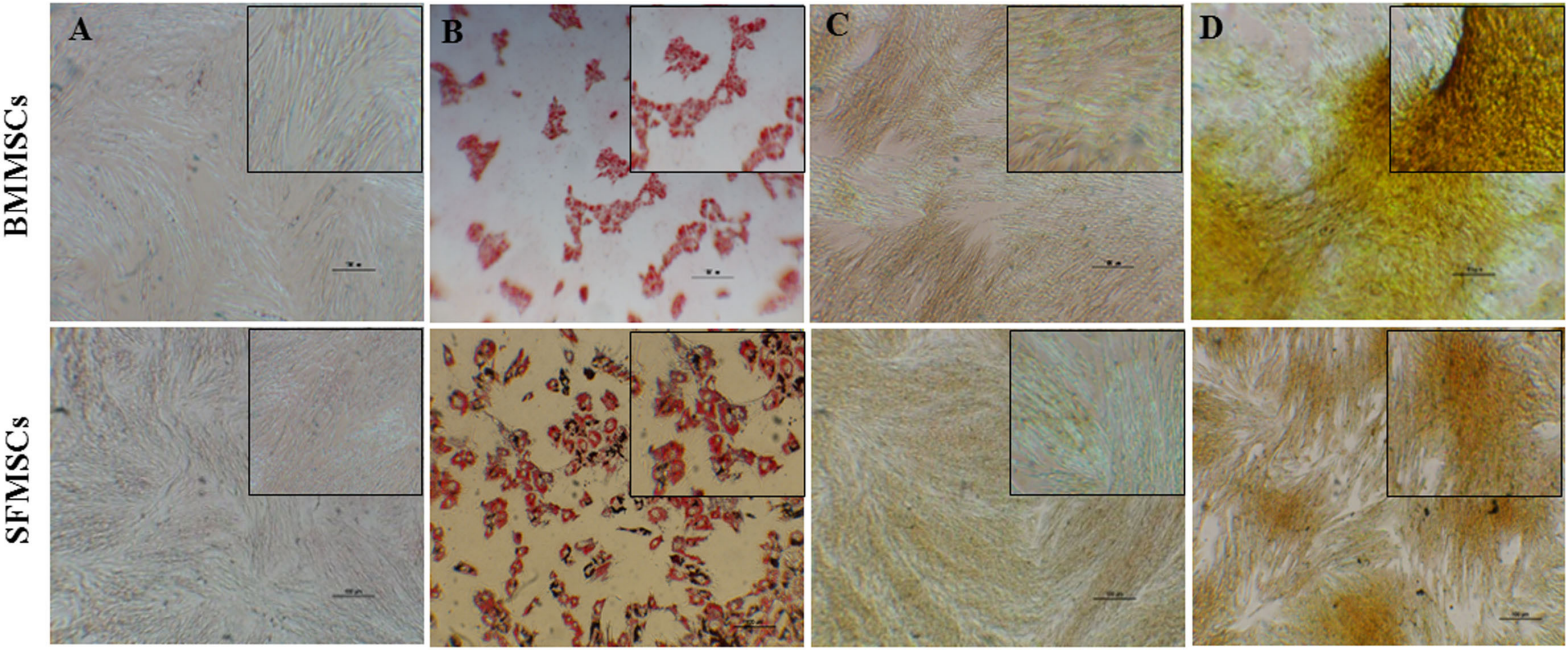
**Representative images of adipogenic and osteogenic differentiation assays**. Bone marrow-derived mesenchymal stem cells (BMMSCs) and synovial fluid-derived mesenchymal stem cells (SFMSCs) were induced to differentiate using lineage-specific media. **(A,B)** Oil Red O staining of cells cultured for 7 days in undifferentiated and adipogenic differentiation medium, respectively. **(C,D)** Alizarin red staining of cells cultured for 14 days in undifferentiated and osteogenic differentiation medium, respectively. Scale bar = 100 μm.

Subjective evaluation of the patterns of differentiation demonstrated that both BMMSCs and SFMSCs undergo differentiation in presence of the lineage-specific induction media. The adipogenic and osteogenic differentiation was demonstrated by the deposition of lipid droplets into the cytoplasm, as visualized by Oil Red O staining and calcium matrix precipitation using alizarin red dye, respectively (Figure 3).

### Chondrogenesis

**Figure 4:**
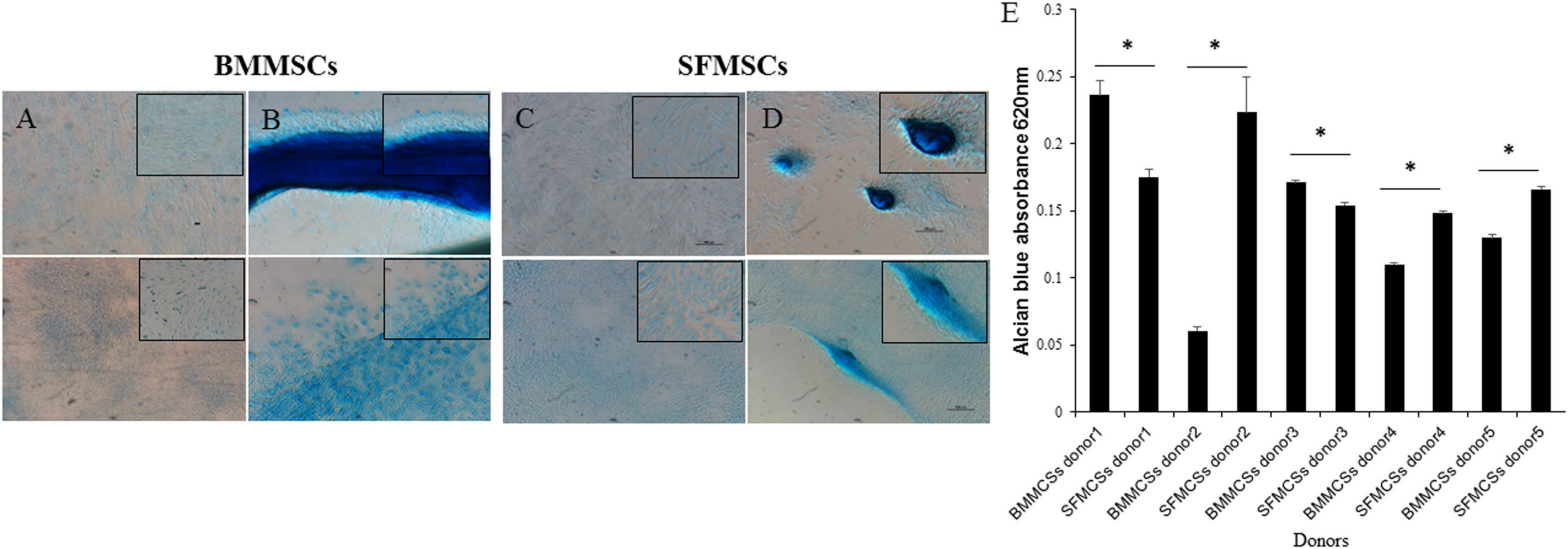
**Representative images of chondrogenic differentiation assays**. Bone marrow-derived mesenchymal stem cell (BMMSCs) and synovial fluid-derived mesenchymal stem cells (SFMSCs) were induced to differentiate using transforming growth factor beta containing media. **(A,B)** Alcian Blue staining of undifferentiated and differentiated BMMSCs, respectively, cultured for 14 days. **(C,D)** Alcian Blue staining of undifferentiated and differentiated SFMSCs, respectively, cultured for 14 days. Scale bar = 100 μm. **(E)** Quantative analyses of glycosaminoglycan (GAG) contents of differentiated BMMSCs and SFMSCs by GAG assay at day 14. Data are presented as means ± SD. Error bars represent the SD. Results are significantly different (*P < 0.001).

Chondrogenic differentiation was demonstrated by the deposition of GAG by Alcian Blue staining. Striking differences were observed, these differences in staining were supported by the variations in the GAG content in each of the differentiated sample (Figure 4).

The GAG content was measured and compared between the BMMSCs and SFMSCs within each donor. SFMSCs of three donors exhibited a significant increase in GAG content compared to the BMMSCs (*P* < 0.0001–0.006). Other two donors, the pattern was reversed and the BMMSCs exhibited a significant increase (*P* < 0.0001–0.003) in GAG content compared to the SFMSCs (Figure 4).

**Figure 5:**
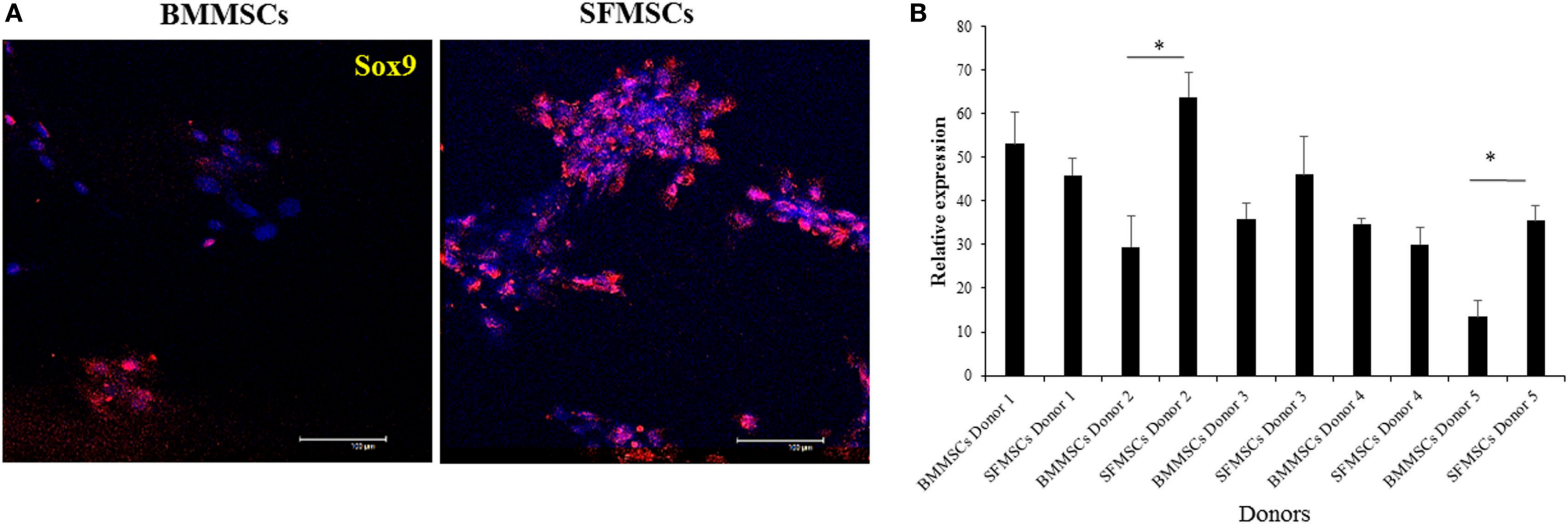
**Representative images to show the expression of cartilage-specific protein marker (SRY-type HMG box9; SOX9) detected by immunofluorescence in cultured bone marrow-derived mesenchymal stem cell (BMMSCs) and synovial fluid-derived mesenchymal stem cell (SFMSCs) from one donor**. **(A)** Cells were stained for SOX9 (red) and DAPI (blue). **(B)** Quantitation of SOX9 expression in BMMSCs and SFMSCs. Results are significantly different (*P < 0.004). Scale bar = 100 μm.

**Figure 6:**
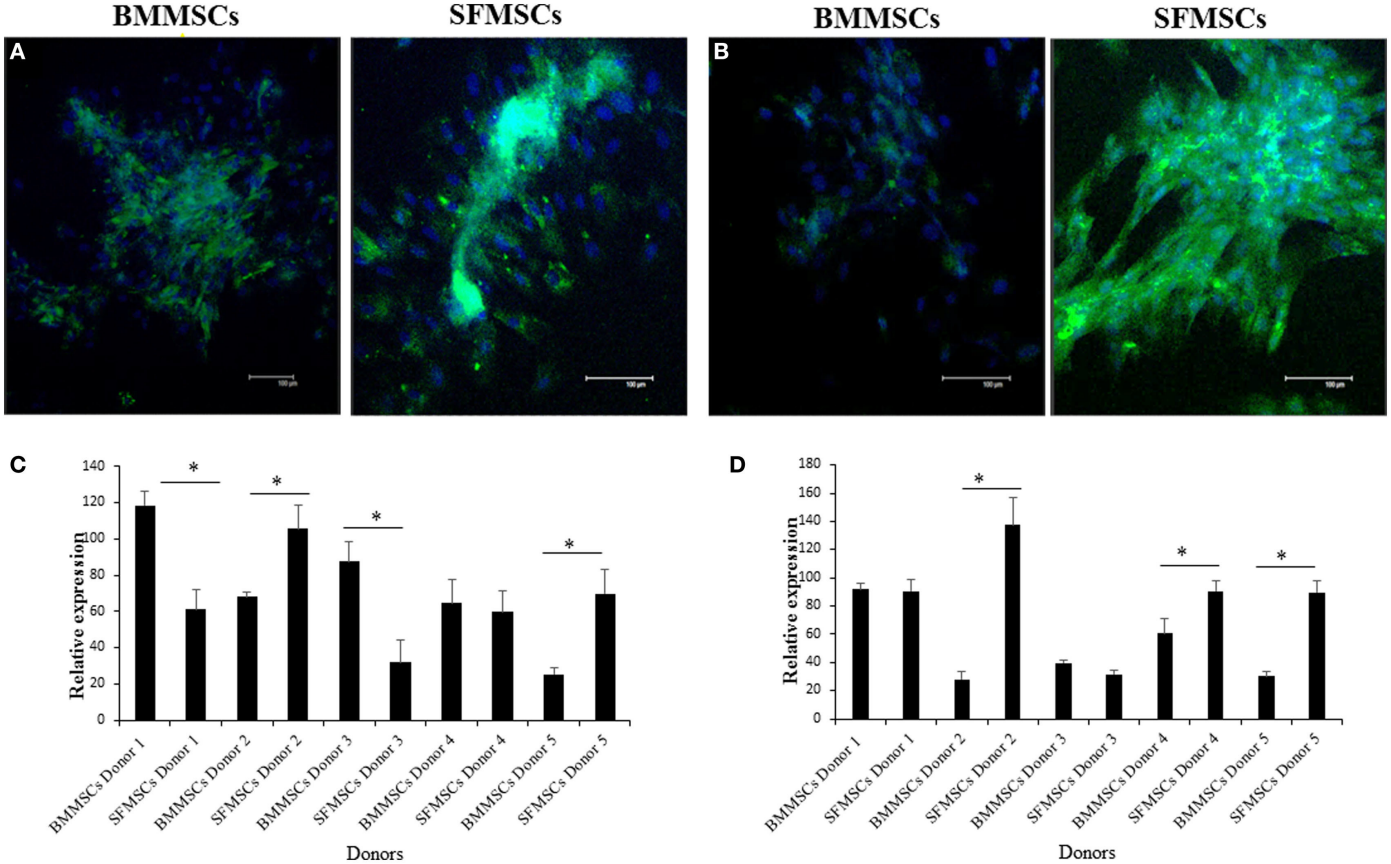
**Representative images to show the expression of cartilage-specific protein markers detected by immunofluorescence in cultured bone marrow-derived mesenchymal stem cell (BMMSCs) and synovial fluid-derived mesenchymal stem cell (SFMSCs) from one donor**. **(A)** Cells were stained for Aggrecan (green) and DAPI (blue). **(B)** Cells were stained for collagen type II (Col II) (green) and DAPI (blue). Quantitation of chondrogenic protein markers, Aggrecan **(C)** and Col II **(D)**, respectively, expression in BMMSCs and SFMSCs. Results are significantly different (*P < 0.004). Scale bar = 100 μm.

The variations in chondrogenesis were further evaluated by assessing the expression of chondrogenic protein markers during differentiation. IF demonstrated the expression of SOX9, Aggrecan, and Col II at 3, 14, and 14 days, respectively, in both cell types (Figures 5 and 6). However, quantitative analyses showed significant variation in their expression levels. The expression levels of all three target proteins were significantly upregulated in SFMSCs compared to BMMSCs from at least two donors. Aggrecan was the only protein marker that was significantly upregulated in BMMSCs cultured from two donors.

## Discussion

Effective cartilage tissue engineering using MSCs mostly depends on the capacity of cells to proliferate, differentiate, and establish cartilage *in vivo*. BM and AD have been the most extensively studied and used sources for obtaining MSCs. To alleviate some of the technical challenges associated with the tissue harvest and properties of MSCs cultured *ex vivo* from BM and AD, recently, SF and the synovium membrane have been identified as alternative sources.

Reports have demonstrated that SFMSCs are multipotent and exhibit properties of adult MSCs; in addition, SF collection is relatively easy and less invasive (17, 18, 26, 29). Studies have reported the isolation, identification, and biological properties of equine SFMSCs and BMMSCs (17, 18, 23, 30). However, a detailed pairwise comparison with respect to the growth, chondrogenic differentiation, and expression of chondrogenic proteins between BMMSCs and SFMSCs from the same donor has not been reported. The impact of donor is particularly critical for autologous treatment regimens and in deciding whether such a cell-based therapy represents the appropriate treatment option for an individual patient. Thus, the purpose of this study was to investigate *in vitro* properties of SFMSCs and BMMSCs generated from five donors with a focus on their cartilage forming potential.

In this study, MSCs adhered to tissue culture flasks and proliferated showing spindle, fibroblast-like morphology that is typical of MSCs. SF harvested from a joint has limited number of progenitor cells and hence requires *in vitro* expansion to generate sufficient numbers of viable MSCs required for the treatment of an injury (18, 31). A linear increase in both BMMSCs and SFMSCs from five equine donors over a period of 8 days (Figure 2), suggested that in presence of an optimal growth medium containing 10% fetal bovine serum, both BMMSCs and SFMSCs are viable and can generate sufficient numbers of MSCs for clinical application (27). Similar expression levels of the three commonly reported CD markers, CD29, CD44, and CD90, in BMMSCs and SFMSCs (Table 3) from all donors further confirmed the “stemness” of these primary cultures. Most importantly, the high level of MHC-II expression suggests that in contrast to the BMMSCs, the SFMSCs may not be used as an allogeneic source of MSCs for therapy; however, *in vivo* investigation is needed.

The potential for trilineage differentiation is yet another important biological property that should always be used to verify the multipotentiality of MSCs. Differences have been reported in these assays in MSCs derived from various sources (14, 19, 30, 32). Consistent with the previous studies, cells from both sources differentiated into adipocytes, osteocytes, and chondrocytes within 7, 14, and 14 days, respectively (14, 18, 32). Of relevance to this study, even though both BMMSCs and SFMSCs from all donors showed the potency to differentiate into chondrogenic lineage, the process exhibited varying degrees. Subjectively, the Alcian Blue staining and quantitatively measured by GAG content, there was a significant upregulation in the chondrogenic potentials of SFMSCs from three out of five donors (Figure 4). On the other hand, the chondrogenic potential of BMMSCs from two donors were upregulated relative to SFMSCs. Thus, results suggest that all SFMSCs are not necessarily superior to BMMSCs in their chondrogenic potential but demonstrate donor-to-donor variation.

Mesenchymal cell differentiation into chondrocytes is a complex process that involves a very well-orchestrated signaling pathway of growth factors (33). In our study, we investigated the changes in expression of specific key markers that coordinate this process in the hope of understanding what makes one MSC culture better suited for chondrogenesis compared to the other. It has been shown that SOX9, a master regulatory factor, regulates a specific set of genes in chondrocytes (34, 35). Aggrecan and Col II are the major components in the articular cartilage, which play an important role in chondrocytes differentiation and chondrocyte–matrix interactions (36, 37). Col II is an important marker for hyaline cartilage, which is valuable when cartilage healing is evaluated *in vivo*. Our results indicate that both BMMSCs and SFMSCs could express SOX9, Aggrecan, and Col II in all primary cultures, however, with varying levels.

The significant upregulation of SOX9 in SFMSCs cultured from two donors, along with a significantly higher GAG content and Aggrecan, suggests that the increased levels of these regulatory proteins may be responsible for their higher chondrogenic potential. Additionally, the significant upregulation of Col II in SFMSCs from three donors suggests that the potential for regeneration of hyaline cartilage is higher in these SFMSCs (Figure 6). Conversely, BMMSCs from other two donors demonstrate significant increase in GAG content coupled with the expression of Aggrecan, but there is no change in the SOX9 expression (Figure 5), suggesting that the chondrogenic potentials in these two donors may be influenced by some other factors unknown at this time. These results suggest that since chondrogenesis is a complex process, it can be affected at multiple points by the variation in expression of chondrocyte progenitor proteins thus leading to significant changes in the process itself. As a result, all SFMSCs need not be superior to BMMSCs but are dependent on the donor.

Another highlight of this study is that we were able to detect the expression of key chondrocyte proteins in equine MSCs, which can prove to be very helpful in future *in vitro* and *in vivo* experiments. In equine medicine, we lack equine-specific antibodies to detect the expression of SOX9, Aggrecan, and Col II limiting us to use those that are raised against mouse or human target proteins (38).

In conclusion, the use of MSCs for cell and tissue regeneration depends on the capability to *in vitro* expand and at the same time maintain their multipotency. Large differences among the donors in growth and response to chondrogenic signals can limit their application. Careful evaluation of various samples from the same donor may improve the clinical outcomes and progress tissue engineering applications. In this study, we did prove our hypothesis and were able to accomplish a pairwise comparison of SFMSCs and BMMSCs in five equine donors. Using *in vitro* molecular and cellular assays, we did identify donors with superior and inferior SFMSCs, which can greatly affect their clinical applications. Further *in vivo* experiments using controlled animal models are necessary to address the performance of SFMSCs, prior to their use in clinical cases.

## Author Contributions

MZ, CC, and MD contributed to study design, data collection, and study execution. NM assisted in data analysis and interpretation; all the authors were involved in the preparation of the manuscript.

## Conflict of Interest Statement

The authors declare that they have no competing interests. A research report of a part of this work was presented at the 2015 North American Veterinary Regenerative Medicine Association (NAVRMA), Monterey, CA, USA.

## Abbreviations

MSCs, mesenchymal stem cells; BM, bone marrow; SF, synovial fluid; BMMSCs, bone marrow-derived mesenchymal stem cells; SFMSCs, synovial fluid-derived mesenchymal stem cells; GAG, glycosaminoglycan; SOX9, SRY-type HMG box9; Col II, collagen type II.
